# Preparation of Poly-Lactic-Co-Glycolic Acid Nanoparticles in a Dry Powder Formulation for Pulmonary Antigen Delivery

**DOI:** 10.3390/pharmaceutics13081196

**Published:** 2021-08-03

**Authors:** Regina Scherließ, Julia Janke

**Affiliations:** 1 Department of Pharmaceutics and Biopharmaceutics, Kiel University, 24118 Kiel, Germany; julia_janke@gmx.de; 2 Merck Healthcare KGaA, 64289 Darmstadt, Germany

**Keywords:** double-emulsion method, dry powder inhalation, antigen release, porous PLGA particles, microfluidics

## Abstract

One of the key requirements for successful vaccination via the mucosa is particulate antigen uptake. Poly-lactic-co-glycolic acid (PLGA) particles were chosen as well-known model carriers and ovalbumin (OVA) as the model antigen. Aiming at application to the respiratory tract, which allows direct interaction of the formulation with the mucosal immune system, this work focuses on the feasibility of delivering the antigen in a nanoparticulate carrier within a powder capable of pulmonary delivery. Further requirements were adequate antigen encapsulation in order to use the characteristics of the particulate carrier for (tunable) antigen release, and capability of the production process for industrialisation (realisation in industry). For an effective particulate antigen uptake, nanoparticles with a size of around 300 nm were prepared. For this, two production methods for nanoparticles, solvent change precipitation and the double emulsion method, were evaluated with respect to antigen incorporation, transfer to a dry powder formulation, redispersion and antigen release characteristics. A spray drying step was included in the production procedure in order to obtain a respirable powder with an aerodynamic particle size of between 0.5 and 5 μm. The dried products were characterised for particle size, dispersibility and aerodynamic behaviour, as well as for immune response and cytotoxicity in cell culture models. It could be shown that the double emulsion method is suitable to prepare nanoparticles (270 nm) and to incorporate the antigen. By modifying the production method to prepare porous particles, it was possible to obtain an acceptable antigen release while maintaining an antigen load of about 10%. By the choice of polyvinyl alcohol as a stabiliser, nanoparticles could be dried and redispersed without further excipients and the production steps were capable of realisation in industry. Aerodynamic characteristics were good with a mass median aerodynamic diameter of 3.3 µm upon dispersion from a capsule-based inhaler.

## 1. Introduction

As a target for vaccination, the respiratory tract offers the advantage that most pathogens enter the body via this pathway. Hence, this route allows direct interaction with the mucosal immune system [1]. This is attractive not only for the efficient prevention of infections such as SARS-CoV-2, which threatens the world with its pandemic spread which started 2019 [2], but also in the course of therapeutic vaccination [3]. Mucosal vaccination is based on the idea that a particulate vaccine formulation is taken up and processed locally. This requires efficient particle uptake by endocytosis or phagocytosis. These processes are clearly size-dependent; the smaller the particles, the better they can be taken up. Hence, it is obvious to use nanoparticles rather than microparticles as antigen carriers for mucosal vaccination, despite the higher amount of antigen per carrier which can be formulated in microparticles. Within the nanometre range, the particles should be large enough not to be drained via the lymphatic route (i.e., larger than 100 nm) and small enough not to be cleared by macrophages to a larger extent. Macrophages generally take up particles of 500 nm and above [4]. For respiratory administration, it is reported that alveolar macrophages predominantly take up particles between 3 µm and 6 µm [5]. Hence, nanoparticulate vaccine carriers should best have a size between 100 nm and 500 nm (possibly up to 3 µm). For the present work, a target size of 300 nm was determined [6]. Nanoparticles can be prepared by a range of different techniques using “top down” technologies, such as milling, or “bottom up” technologies, such as precipitation, ionic gelation or double emulsion/solvent evaporation techniques [7]. All these processes take place in liquid phase and result in a suspension of nanoparticles in the respective dispersion media, mostly an aqueous system. Nonetheless, physical and thermal stability may be greatly increased when the product can be dried. A drying process shall not harm the antigen, and it also has to retain the nanoparticle being formulated in the preceding step. This comprises retention of primary nanoparticle size and redispersion capability. Spray drying (or freeze drying) is a feasible technique, but further efforts need to be made to stabilise the nanoparticles and allow redispersion. If a nanoparticle suspension is spray dried without excipients, the nanoparticles will probably aggregate to a great extent due to their large surface. Depending on the carrier material, they may also coalesce to larger, undefined material and completely lose their initial small size during wet storage or drying without excipients. This is detrimental for nanoparticulate uptake of antigen and also with respect to pulmonary delivery, where an aerodynamic particle size between 0.5 µm and 5 µm is aimed at [8]. To facilitate redispersion, the nanoparticles are hence embedded into a microparticulate matrix during spray drying, resulting in Nano-in-Microparticles (NiMs). With this, nanoparticle agglomeration and aggregation are prevented and the product is easier to handle. If the matrix is water soluble, such as a carbohydrate, the matrix can dissolve upon water contact (e.g., deposition on the wet mucosa) and release the embedded nanoparticulate vaccine carrier, which can then be taken up.

Ideally, a polymer for vaccination should be degradable in the body to allow the release of antigen and clearance of the drug. It should be biocompatible, of low toxicity and without intrinsic immune response. It needs to be water insoluble to allow particulate uptake, but sufficiently soluble (under non-physiological conditions) in (non-chlorinated) aqueous/organic solvents to allow formulation. The polymer needs to be compatible with proteins and has to be solid at room temperature and up to about 40 °C to allow formulation and storage as a dry powder. Poly-lactic-co-glycolic acid (PLGA), which was used as the model polymer in this study, is a synthetic polymer, which is only soluble in organic solvents. The polymer can be degraded hydrolytically over time [9], resulting in the monomers lactic acid and glycolic acid. Degradation is a function of polymer length (molecular weight) and composition and may take up to several months, making it suitable for sustained-release dosage forms [10]. The shortest degradation time is described for a 50:50 (lactic acid to glycolic acid) polymer, which is used in this work. The polymer can further be functionalised [11,12]. It has been proved that nanoparticles made of, e.g., PLGA can be used as carriers for the antigen to increase the elicited adaptive response by means of increased antigen uptake, processing and presentation [13,14]. PLGA particles have been shown to be effective vaccine carriers upon respiratory administration [15,16]. However, due to their slow degradation, PLGA particles appear to be more suitable for sustained release or depot formulations [10], whereas for vaccination, an immediate and sustained presence of the antigen is required. It had been shown that a more rapid release from PLGA derivatives was associated with better immune response [17], but the use of novel polymers goes along with safety issues. Thus, the well-known PLGA was used as model and nanoparticle preparation was modified to allow less-sustained release of the antigen.

The aim of this work was to design a formulation which can be administered by dry powder inhalation, and which can be prepared using industry-realisable and scalable processes.

## 2. Materials and Methods

Nanoprecipitation

For solvent-change nanoprecipitation, 1.6% (m/V) polylactic-co-glycolic acid (PLGA, Resomer 503 H, Evonik, Darmstadt, Germany) was dissolved in a mixture of 9 + 1 acetone (J.T.Baker, Deventer, The Netherlands) and methanol (J.T.Baker, Deventer, The Netherlands). This organic phase was added dropwise in an aqueous phase containing 0.5% (*w*/*w*) HPMC (Metolose 60 SH 50, Shin-Etsu, Tokyo, Japan), or other stabilisers such as 0.1% Polysorbate 80 or 0.1% Poloxamer 188 in ultrapure water. The mixture was stirred with an Ultra-Turrax (IKA, Staufen, Germany) at 9500 rpm. When the addition of organic phase was completed, the speed was raised to 13,500 rpm for three minutes. Afterwards, the dispersion was stirred with a magnetic stirrer (IKA, Staufen, Germany) at 600 rpm in order to evaporate the organic solvents. After the evaporation process, the dispersion was sonicated in an ultrasonic bath (Sonorex Super RK 106, Bandelin electronic GmbH, Berlin, Germany) for 10 min in order to disperse agglomerates. To determine the suitability of micromixers (interdigital slit mixer and impinging jet mixer, IMM, Mainz, Germany), the two phases were pumped by HPLC pumps (Knaur, Berlin, Germany) into the respective mixing device. The resulting nanosuspension was collected in a glass and was stirred to remove the organic solvent as described above. To load the antigen to the particles, ovalbumin (Sigma, St. Louis, MO, USA) as the model antigen was adsorbed to the particle surface. An ovalbumin stock solution (2 mg/mL) was produced by dissolving ovalbumin in phosphate buffered solution at pH 7.4. For adsorption, the nanoparticle dispersion was mixed with the ovalbumin stock solution at a ratio of 2 + 1 (dispersion + ovalbumin stock solution) and shaken for 4 h at 37 °C prior to spray drying.

Double emulsion method

For NP preparation via the double emulsion method, 2.5% (m/V) PLGA was dissolved in ethyl acetate (Merck, Darmstadt, Germany) as organic phase. This organic phase was homogenised for two minutes at 20,500 rpm by Ultra-Turrax (IKA, Staufen, Germany) with a first hydrophilic phase containing 4% (m/V) ovalbumin (Sigma-Aldrich, St. Louis, MO, USA) in phosphate buffered solution of pH 7.4, resulting in a W/O emulsion. Afterwards, a second hydrophilic phase containing 5% (m/V) PVA (Mowiol 4-88, Hoechst, Frankfurt, Germany) dissolved in ultrapure water was added and homogenised for two minutes. The produced W/O/W-emulsion was transferred into a stabilising solution with 1% PVA in water and stirred with a magnetic stirrer (IKA, Staufen, Germany) at 400 rpm in order to harden the produced particles and to evaporate the organic solvent. Afterwards, the particles were washed to remove free ovalbumin. For this, the dispersion was centrifuged at 14,000 rpm (Centrifuge 5430 R, Eppendorf, Hamburg, Germany), leading to a clear supernatant. The supernatant with free ovalbumin was removed and replaced by a fresh 1% (m/V) PVA solution (and in some experiments 0.01% (m/V) l-leucine) for redispersion of the nanoparticles.

Porous nanoparticles

Porous particles were produced by the double emulsion technique. Here, the first hydrophilic phase was prepared by dissolving 4% (m/V) ovalbumin as well as trehalose (5% to 50% (m/V), British sugar plc, Peterborough, UK) in phosphate buffered solution with pH 7.4. Preparation of W/O and W/O/W emulsion and washing was performed as described above. The supernatant was replaced by a solution containing 1% (m/V) PVA in water for redispersion.

Dry powder formulation by spray drying

The nanoparticle dispersion was spray dried using the Mini-Büchi B-290 (Büchi, Flawil, Switzerland) equipped with a two-fluid nozzle and a high-performance cyclone. No further matrix excipients were added prior to spray drying. Drying was performed at an inlet temperature of 100 °C and an outlet temperature of about 42 °C in order to not melt the polymer.

Particle size distribution and morphology

Nanoparticle size was characterised by dynamic light scattering (Zetasizer, Malvern Instruments, Malvern, UK) before spray drying and after redispersion of the dry powder in water to determine the particle diameter and polydispersity index (PDI). For this, every sample was assayed in triplicate. Particle size of the dry powder after spray drying was characterised by laser diffraction using dry dispersion at 3 bar (Helos with Rodos module, Sympatec GmbH, Clausthal-Zellerfeld, Germany). To visualise particle size and morphology, SEM pictures of the respective formulations were taken (Smart SEM Supra 55VP or Zeiss DSM 940, Zeiss, Oberkochen, Germany).

Antigen content

For antigen quantification, the micro-BCA assay (Thermo Scientific, Rockford, IL, USA) with an OVA calibration was used. When antigen content of intact nanoparticles was determined, PLGA particles were degraded in 0.1N NaOH and samples were neutralised with 0.1 N HCl prior to protein quantification.

Dispersion behaviour and aerodynamic characterisation

**Figure 1:**
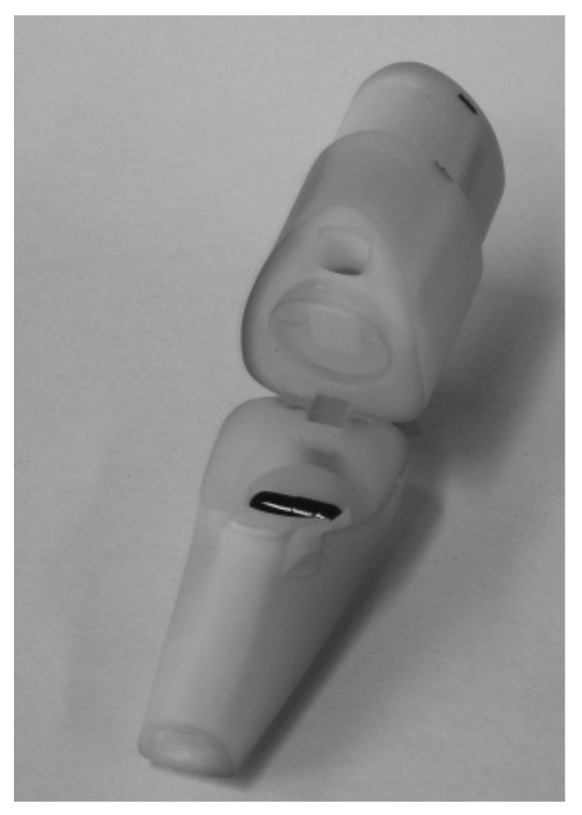
Unihaler.

**Figure 2:**
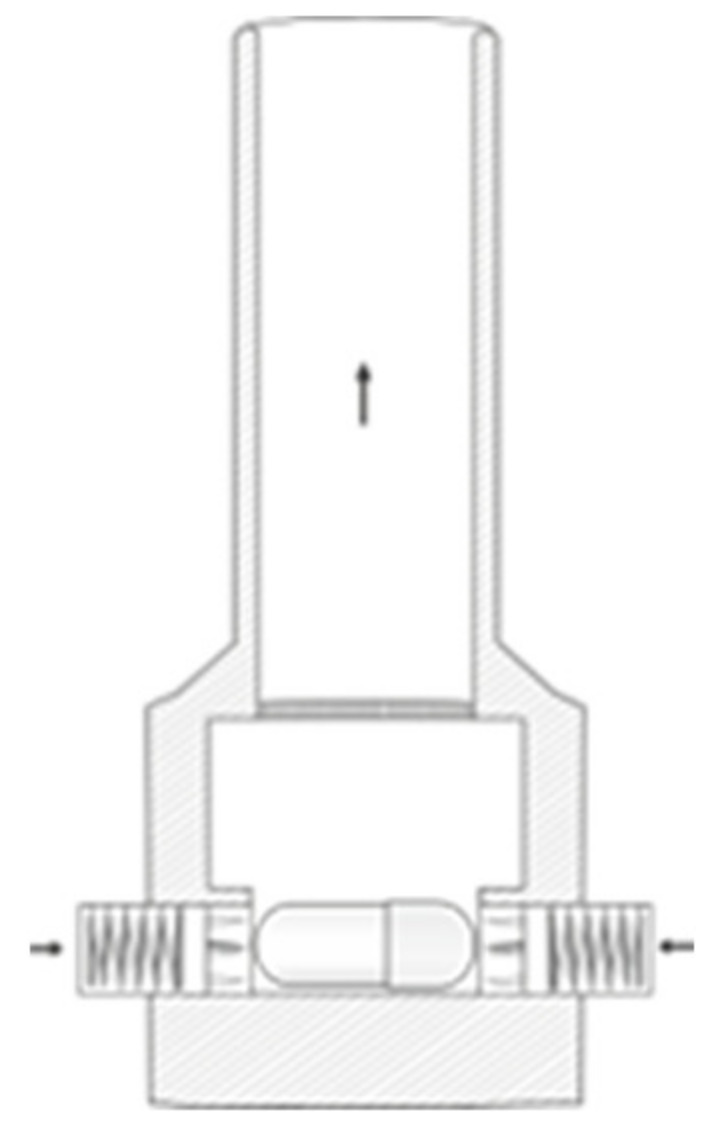
Schematic of the Cyclohaler.

For device dispersion experiments and aerodynamic characterisation of the formulations, two capsule-based inhalers, the Unihaler (Figure 1) and the Cyclohaler (Figure 2), were used.

Capsules (HPMC capsules, size 3, Qualicaps Europe, Alcobendas, Spain) were filled manually with 20 mg of the respective powder. Particle size distribution upon device dispersion was measured with the INHALER module (Helos, Sympatec GmbH, Clausthal-Zellerfeld, Germany) at the respective flow rate, creating a pressure drop of 4 kPa over the device (59 L/min for the Unihaler and 100 L/min for the Cyclohaler). Aerodynamic characterisation was performed with the Next Generation Pharmaceutical Impactor (NGI, Copley Scientific, Nottingham, UK). One capsule was used per run. The measurements were performed in a conditioned environment at 21 °C and 45% rH. All stages were coated with a mixture of propylene glycol and isopropanol 50:50 to minimise particle bounce. Deposited powder was dissolved in 0.1 M NaOH. After neutralisation with 0.1 M HCl, the samples were analysed for ovalbumin content with a Micro BCA Protein Assay Kit (Thermo Scientific, Rockford, IL, USA). As reference, 20 mg of powder was dissolved and analysed with the BCA assay. All samples were analysed four times. The fine particle fraction and mass median aerodynamic diameter of the delivered dose were calculated using the Copley Inhaler testing data analysis software (Citdas, Version 3.10, Copley, Nottingham, UK).

Antigen release

For release studies, 10 mg of the spray dried powder was weighed in 2 mL centrifuge tubes in duplicate for every time point. Afterwards, 1 mL of release medium (phosphate buffered solution at pH 7.4 or pH 5.5, prepared according to Ph. Eur. 7.0) was added. Samples were agitated in a water bath with 40 rpm at 37 °C. At predefined time points over 24 h, the respective samples were removed and centrifuged at 14,000 rpm for 10 min to separate any particles from the supernatant. The supernatant was analysed for ovalbumin content. All samples were analysed four times.

Formulation toxicity by MTT assay and endotoxin content

Formulation toxicity in vitro on Calu-3 cells and endotoxin content by an LAL test were assessed. For cytotoxicity testing, 3 × 10^4^ cells were used per well and the test was performed after 3 days of cell growth. In every well, 200 µL of sample was added and incubated for 4 h at 37 °C and 5% CO_2_. The samples contained spray dried formulation suspended in Hanks balanced salt solution (HBSS) with 30 mM HEPES, a negative control (HBSS) or a positive control (5 mM SDS in HBSS). Every sample was determined 4-fold. After 4 h of incubation, the samples were replaced by 25 µL MTT solution and incubated for 2 h. Afterwards, 100 µL lysis solution (5% SDS in 50:50 DMF:water with pH 4.7) was added into each well. Then, the absorbance of every well was determined by using a plate reader (Spectra Thermo Reader with Software easyWINfitting V6.0a, Männedorf, Switzerland) at 570 nm (reference wavelength of 690 nm). By using the absorbance of the negative and positive control, the cell viability in each well was calculated.

For the determination of endotoxin content, 20 mg of spray dried formulation was redispersed with 5 mL LAL reagent water (Acila, Weiterstadt, Germany) and lightly shaken for 60 min. Afterwards, the particles were removed by centrifugation, 45 min at 7800 rpm, and the endotoxin content in the supernatant was determined by using LAL reagent Limusate^®^ (sensitivity 0.03 E.U./mL, Waku Chemicals, Richmond, VA, USA). Therefore, different dilutions of the supernatant were prepared, and the LAL reagent was added. After 60 min of motionless incubation at 37 °C, the samples were tested for gelling. From sample dilution and sensitivity of reagent, the endotoxin content was determined semi-quantitatively. These tests were performed to ensure the prepared nanoparticles and resulting dry powders were not harmful to the cells and did not induce an unspecific immune response during further in vitro testing.

Storage stability

Storage stability of the dry powder was assessed over time (0, 1, 3 months, 6 months, ongoing) at ambient conditions (21 ± 1.3 °C and 32 ± 3% rH). At the respective time points, a sample was analysed with respect to redispersion and nanoparticle size, protein content, dry powder particle size, dispersion and aerodynamic characteristics.

## 3. Results and Discussion

The absence of chlorinated solvents was one prerequisite for the formulation of PLGA nanoparticles due to toxicological considerations. Therefore acetone, methanol and ethyl acetate were used as solvents for PLGA, unlike in many other studies which use methylene chloride. An advantage of acetone and methanol is their fast evaporation, which eases removal from the formulation. Ethyl acetate is more challenging to remove and is further the most toxic solvent of the aforementioned. Hence, special attention needs to be given to possible residues in the final formulation. Both evaluated formulation processes, nanoprecipitation and the double emulsion technique, resulted in solid nanoparticles of the targeted size range with an acceptable low-size distribution as seen from the PDI (Table 1). It had been assessed earlier how preparation parameters influence the particle characteristics of the resulting particles [18], and parameters had been optimised to achieve the targeted size. For nanoprecipitation, different stabilisers had been screened (polysorbate, Poloxamer 188, HPMC and PVA), with PVA resulting in the smallest nanoparticles below the target size. HPMC, being a macromolecule of moderate surface activity, was able to increase the size concentration-dependently. As HPMC is also favourable if the product is to be dried afterwards, this has been used for nanoprecipitation. For the double emulsion method, HPMC could not be used as the concentration needed for stabilisation, resulting in an unmanageable viscosity. Here, the nanoparticle size was governed by the emulsion droplet size and as such by the homogenisation, whereas an efficient stabiliser (namely PVA) was required to quickly cover interfaces and prevent droplets from growing.

Nanoprecipitation is a process which can easily be automated and may run in a semi-continuous mode by the use of a central mixing device which is constantly supplied with the polymer solution and non-solvent by two pumps providing constant flow rates [19,20]. This has been evaluated in this study for an interdigital slit mixer and an impinging jet mixer. The advantage of an impinging jet mixer is that precipitation takes place externally. With this, the device is less prone to blocking. For the interdigital slit mixer, resulting particles were comparable to the manual process, but showed a broader distribution (PDI of 0.3) and tended to block the mixing chamber. Initial experiments with the impinging jet mixer resulted in smaller particles of about 100 nm (PDI 0.1), which would be too small for the intended use as an antigen carrier, but the size was shown to be tuneable by parameter optimisation [18]. Industrialisation of a micromixing setup can be performed by “numbering up” (simultaneous use of many micromixing devices) without changing the dimensions of the individual mixing element. The micromixers were used at a flow rate of 24 mL/min, resulting in a solid output of 424 mg/min. For the double emulsion method, focused ultrasound was evaluated by a partner and was found to be a suitable preparation technique, which can be scaled to industrial size [21]. Utilising the same equipment, namely a focused ultrasound system (S220x, LGC-KBioscience, Teddington, UK), batch sizes between 1 mg and 2.5 g could be prepared uniformly.

One important difference in the two preparation methods (precipitation vs. emulsion) is the incorporation of antigen. Nanoprecipitation results in solid PLGA particles, where the antigen needs to be adsorbed to in a second step. If the antigen is present in the non-solvent during precipitation, it might be incorporated into the polymer matrix in parts but will mostly be present at the surface due to its surface activity. Adsorption on the surface will further be hindered by stabiliser molecules which also adsorb to the interface. Hence, comparably low amounts (4% *w*/*w* in this study) were associated with the particles and release of the antigen is predominantly guided by desorption and diffusion. This leads to soluble antigen being present around the particles as soon as they are deposited on wet mucosal surfaces. This effect is unwanted as particulate uptake of the antigen is a prerequisite for local processing. For this reason, nanoprecipitation is not the best technique for the formulation of antigen-carrying nanoparticles with sustained release. If solvent-change precipitation can be used to prepare nanocapsules by coating antigen crystals, the product could have superior characteristics. This approach was not followed further in this work, but it focussed on the double emulsion method, which incorporates the antigen being dissolved in the inner phase of the primary emulsion into the polymer matrix. Here, loading capacities of 10% and a size of 270 nm with narrow distribution were reproducibly achieved (Table 1).

**Figure 3:**
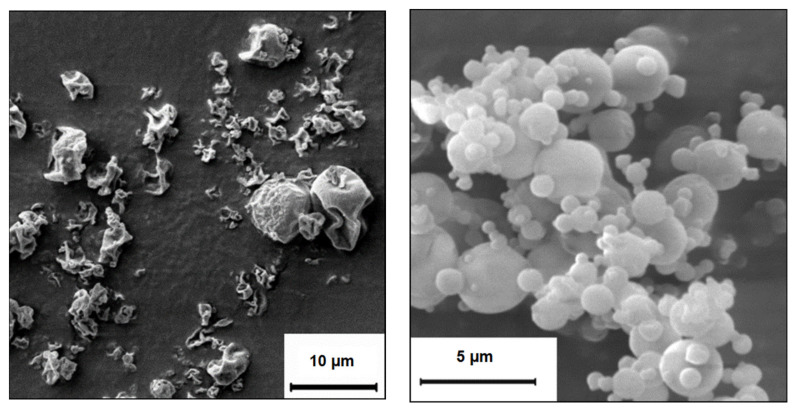
SEM pictures of spray dried powder. **Left**: PLGA nanoparticles loaded with OVA prepared by solvent change evaporation and 0.5% HPMC as stabiliser/matrix. **Right**: PLGA nanoparticles with OVA prepared by the double emulsion technique and 1% PVA as stabiliser/matrix.

The formulations were transferred to a dry powder by spray drying to increase storage stability and allow direct application of the dry powder by inhalation. Here, only HPMC and PVA were suitable as stabilisers of the nanosuspension as the stabiliser cannot be removed completely prior to spray drying. If a liquid surfactant, such as Polysorbate 80, was used as stabiliser, the resulting product after spray drying was highly aggregated and sticky, and hence unsuitable for dry powder dispersion. HPMC and PVA as stabilisers resulted in a dry powder with good bulk characteristics and dispersion behaviour without the need of further matrix components. During spray drying, the nanoparticles were incorporated into the polymeric stabiliser, being present in excess and forming microparticles (Figure 3). The particle size of the spray dried powder is governed by atomisation and solid content of the feed liquid [22].

**Figure 4:**
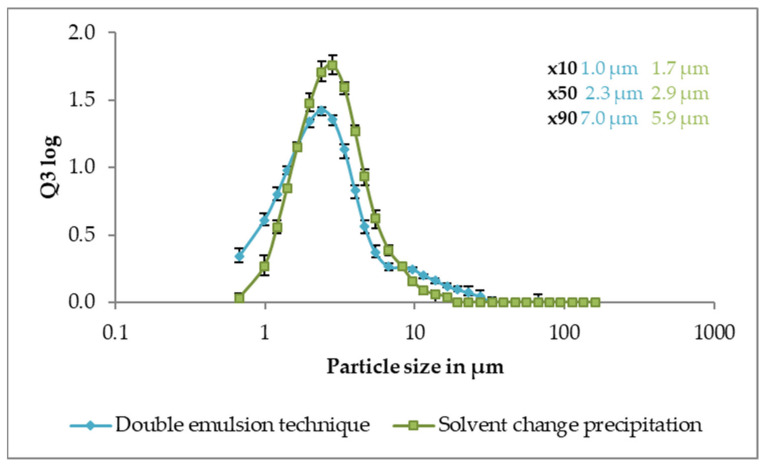
Particle size distribution of spray dried powders with nanoparticles prepared via solvent change precipitation (green) or double emulsion technique (blue) as measured by laser diffraction (3 bar pressurised air dispersion). Data is average of n = 7, error bars show min–max.

Both dry powder formulations were easily redispersible in water and nanoparticle size was unchanged (data not shown). The powders were well dispersible in dry air and resulting particle size distribution was monomodal with a mean size below 3 µm (Figure 4).

**Figure 5:**
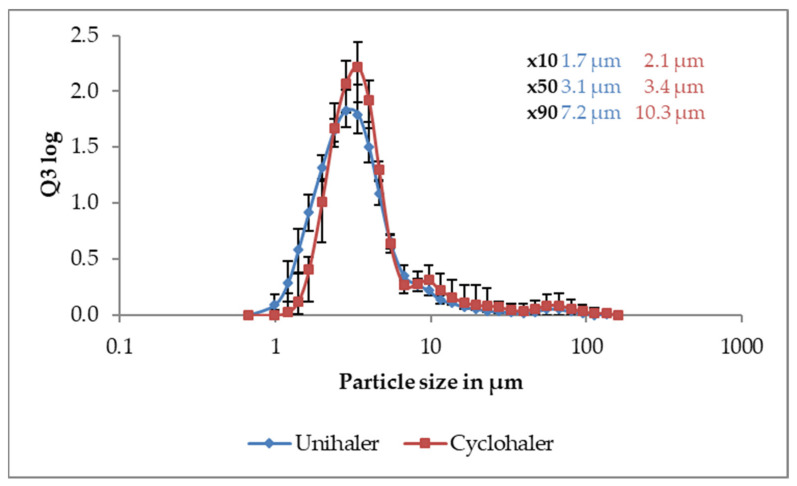
Particle size distribution of spray dried powder with nanoparticles prepared via double emulsion technique dispersed from the Cyclohaler (red) or the Unihaler device (blue) as measured by laser diffraction. Data is average of n = 10, error bars show min–max.

As nanoparticles prepared by the double emulsion technique reveal preferable characteristics for vaccination in terms of antigen incorporation, this formulation approach was used for more detailed characterisations. When the dry powder was dispersed from a capsule-based device, particle size distribution is almost identical to 3 bar pressurised air dispersion as seen in Figure 5. This shows that the powder is excellently dispersible to individual primary microparticles by both tested devices. A slight difference between the two tested devices can be seen, which is probably due to differences in device setup, but this effect does not translate to the aerodynamic assessment.

**Figure 6:**
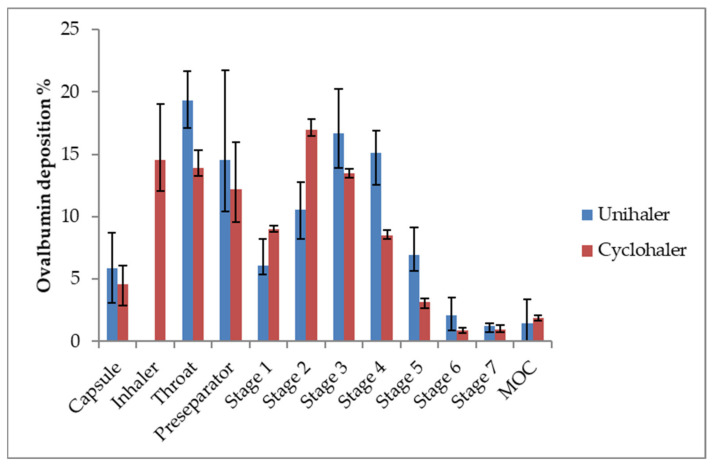
Deposition profile of the NGI of spray dried powder with nanoparticles prepared via double emulsion technique dispersed from the Cyclohaler (red) or the Unihaler (blue) device. n = 3, error bars show min–max.

Aerodynamic assessment with the NGI revealed a good distribution profile in the NGI (Figure 6), resulting in a fine particle fraction of about 50% of the loaded dose. Nonetheless, it could be observed that a proportion of the formulation remains in the capsule and the device, which is unwanted. Moreover, it would be favourable to maximise FPF further. An excipient which could decrease capsule and device retention and could maximise FPF is leucine, which has already been assessed as a dispersion modifier and surface coating in dry powder inhalation formulations [23,24].

An addition of 0.01% leucine in the hydrophilic stabiliser phase prior to spray drying resulted in a slightly decreased particle size of the spray dried powder and lower capsule/device retention, but did not improve FPF further (Table 2).

One important measure for PLGA as a sustained-release polymer is the release of the antigen from the formulation over time. Upon uptake of PLGA nanoparticles, they are routed into intracellular compartments which are acidified [25]. Here, they need to release the antigen, which should then leave the endosome to be processed and presented. Hence, release studies at a reduced pH of 5.5 representing the endosome [26] are relevant to estimate the possibility of the antigen to be released at these conditions.

**Figure 7:**
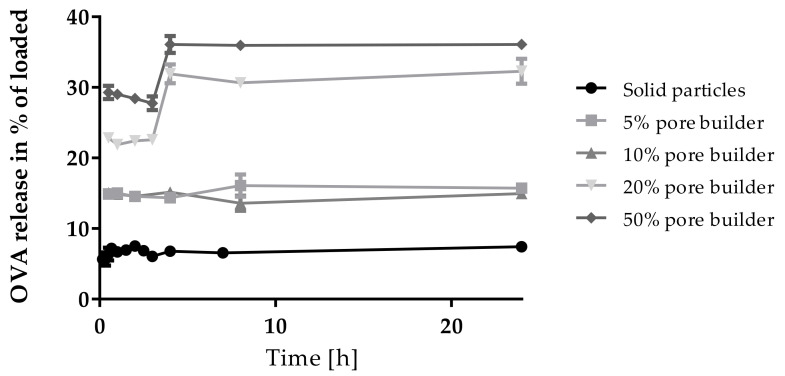
Release of OVA from OVA-loaded PLGA nanoparticles produced by double emulsion technique (without (solid) and with different % amounts of pore builder) in % of loaded OVA over 24 h. n = 3, error bars are standard deviation.

**Figure 8:**
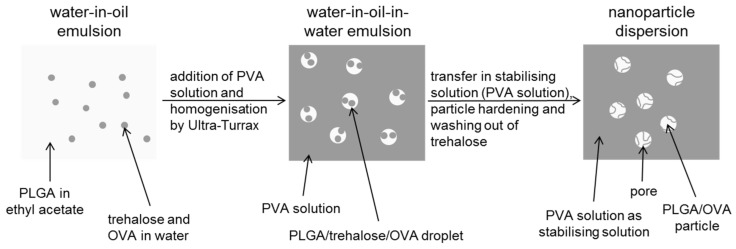
Preparation steps for the formulation of porous PLGA nanoparticles.

The immune effect of solid antigen-loaded PLGA particles is limited, as shown in in-vitro models [17], and this finding goes along with a low release of the antigen over the first 24 h (Figure 7, black line). To increase antigen release, porous nanoparticles were developed (Figure 8).

Depending on the percentage of pore builder, an increased amount of OVA was released over 24 h (Figure 7). Nonetheless, the release profile remained similar: antigen release was dominated by an initial burst release followed by a plateau. This is due to the antigen being incorporated closely to the surface of the particles as PLGA will not degrade that fast. If release is followed over a much longer time period, PLGA degradation leads to further release, but for a vaccination setup, antigen released over the first 24 h after uptake is most interesting [27]. With increasing amounts of pore builder, loading capacity of the nanoparticles decreased and a maximum of 1498 µg OVA released per 100 mg of polymer was observed when pores were formed from 10% trehalose in the first hydrophilic phase (Table 3). When compared to the data from [17], even a low pore builder concentration of 5% should be sufficient to increase the release to be sufficient to provoke a sound immune response while maintaining a good loading capacity.

Endotoxin contamination can play an important role in immunological setups, as endotoxins can activate the immune system themselves, leading to false positive results in in vitro experiments and to unforeseeable reactions in vivo. Endotoxin content of the spray dried powders was between 0.37 and 0.75 I.U./mg depending on the formulation components, which is in an acceptable range [28]. Cell culture experiments revealed no acute toxicity of the dry formulation in concentrations up to 20 mg powder/mL (data not shown). If biocompatibility should be shown, the assay would need to be performed for longer. However, the general biocompatibility of PLGA is well known. The purpose of this study was to assess possible acute toxic effects, which would exclude the prepared particles from further in vitro and in vivo examinations.

Stability of the dry powder formulation (solid PLGA NP prepared by double emulsion) was assessed for storage at room temperature. Particles were redispersible after storage and mean nanoparticle size increased marginally (264 nm ± 5 nm vs. 278 nm ± 9 nm) due to a slightly increased PDI (0.109 vs. 0.180). Dry powder dispersion and aerodynamic characteristics, which started at a lower level than the batches tested before, were improved over storage, resulting in a higher FPF of 34% after 3 months compared to 27% directly after preparation.

## 4. Conclusions

As the double emulsion technique allowed the removal of non-incorporated OVA prior to spray drying with a washing step, and with this, minimisation of free OVA in the formulation was possible, this preparation technique is preferred for the preparation of nanoparticulate PLGA systems for mucosal vaccination. The primary nanoparticles of a size between 250 nm and 300 nm incorporated about 10% OVA as model antigen. Particles were formulated to a dry powder which is easily dispersible by capsule-based dry powder inhalers, resulting in a good fraction of fine particles < 5 µm (aerodynamic particle size) which can enter the lung upon oral inhalation. Immunological evaluation of PLGA particles showed that they are capable of antigen delivery and can provoke an antigen-specific immune response.

## Figures and Tables

**Table 1:** Characteristics of solid nanoparticles produced with the different techniques (manual process).

	Solvent Change Precipitation	Double Emulsion
**Mean particle size**	250 ± 7 nm	270 ± 20 nm
PDI	0.11 ± 0.02	0.13 ± 0.03
**Redispersibility**	good	good
OVA loading	by adsorption	incorporation in first hydrophilic phase
**Loading capacity (OVA)**	4% (*w*/*w*)	10% (*w*/*w*)
SEM image of nanoparticles	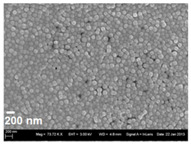	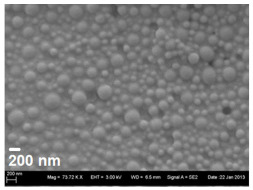

**Table 2:** Mean particle size from laser diffraction measurements (3 bar), mass median aerodynamic diameter (MMAD) and fine particle fraction (FPF) < 5 µm from aerodynamic characterisation in the NGI utilising two capsule-based devices for different dry powder formulations (average of n = 3). n.d. = not determined.

Formulation	Mean Particle Size (x_50_, 3 Bar)	Cyclohaler		Unihaler	
		FPF	MMAD	FPF	MMAD
solvent change precipitation, HPMC	2.3 µm	n.d.	n.d.	n.d.	n.d.
double emulsion, PVA	2.9 µm	51%	3.3 µm	49%	3.1 µm
double emulsion, PVA + 0.01% leucine	2.6 µm	45%	3.5 µm	42%	3.2 µm

**Table 3:** Effect of trehalose addition on OVA loading capacity and OVA release.

Formulation	Loading Capacity, %	Released OVA after 24 h Normalised to 100 mg Polymer, µg
50% trehalose	3.6	1024
20% trehalose	4.6	1120
10% trehalose	9.8	1498
5% trehalose	8.6	1283
solid particles (no pore builder)	11.5	789

## Data Availability

Not applicable.
